# Editorial: Interview With the Translational Apparatus: Stories of Intriguing Circuits and Mechanisms to Regulate Translation in Bacteria

**DOI:** 10.3389/fmicb.2021.707354

**Published:** 2021-06-18

**Authors:** Anna Maria Giuliodori, Stefano Marzi

**Affiliations:** ^1^School of Biosciences and Veterinary Medicine, University of Camerino, Camerino, Italy; ^2^Université de Strasbourg, CNRS, Architecture et Réactivité de l'ARN, UPR9002, Strasbourg, France

**Keywords:** bacterial translation regulation, antibiotics mechanisms, stress responses, sRNA, translation elongation pauses, ribosome rescue mechanisms

Bacteria can adapt promptly to environmental changes using regulatory switches which work at both transcriptional and post-transcriptional levels. Translational control offers a rapid and precise response to different stresses insofar as the signals are processed to get immediate proteome remodeling. The players of this regulation are mRNAs, tRNAs, nucleic acid binding proteins, regulatory sRNAs, and the ribosome itself. Depending on the state of the cell, they can establish different regulatory networks via direct interactions, structural changes, and RNA modification states. Translation initiation, involving ribosome loading on the mRNA start codon is most often the step subjected to regulation. The accessibility of several mRNA signals, including Shine-Dalgarno (SD) sequences, A/U rich tracts recognized by ribosomal RNA chaperone bS1, and the start codon, can be regulated in response to environmental clues, interactions with proteins, sRNAs or metabolites (Geissmann et al., 2009; Giuliodori et al., 2010; Kortmann and Narberhaus, 2012; Milón and Rodnina, 2012; Duval et al., 2013, 2015; Gualerzi and Pon, 2015). Nevertheless, translation rates are also modulated at the elongation, termination and ribosome recycling steps (Rodnina, 2018; Burroughs and Aravind, 2019; Tollerson and Ibba, 2020). Furthermore, an additional layer of regulation is exerted by the delicate balance between RNA stability (mRNAs and sRNAs) and translation activity or by the coupling of transcription and translation, which occurs with specific mechanisms and to different extents in different bacterial genera.

This Research Topic represents a collection of articles, including both original researches and reviews, illustrating the molecular diversity of bacterial translation regulation and describing the various techniques employed for its study. Microbiology, genetics, molecular and structural biology or deep sequencing analyses of translation rates and pausing/arresting sites are some of the approaches outlined. The articles are covering translation regulation at different levels.

The review by Cheng-Guang and Gualerzi describes three cellular responses to stresses in which the ribosome directly participates as sensor and/or as target. Different paralogs of ribosomal proteins and of an enzyme involved in initiator fMet-tRNA_fMet_ biosynthesis are involved in zinc homeostasis and in the response to zinc starvation. The (p)ppGpp alarmones are produced in response to the binding of an uncharged cognate tRNA to the A-site of stalled ribosomes under a variety of stress conditions, including starvation for an essential amino acid. Notably, (p)ppGpp can target the assembly of translation initiation complexes in a mRNA dependent way. Finally, a combination of mRNA structures and RNA chaperone proteins orchestrates translation during cold shock.

Pourciau et al., describes the role played in *Gammaproteobacteria by* CsrA and its homologs. CsrA is the global regulator of carbon metabolism in the stationary phase, also coordinating virulence and motility. This RNA-binding protein establishes regulatory loops including its own autoregulation. To do this CsrA operates at different levels using different mechanisms, including translation repression or activation, promotion of transcription termination, and RNA stabilization.

The article from Maksimova et al., reports a systematic approach, including kinetics and thermodynamics studies, to investigate the effects on different stages of protein synthesis of Amicoumacin A, an antibiotic that targets the 30S ribosomal subunit. The results provide insight on its original mechanism of action, involving stabilization of the mRNA and the 30S initiation complex.

Translation regulation does not finish with the initiation step. Samatova et al. discuss the surprising world of translation pausing, reviewing the most recent advancements in this field. The pauses of elongating ribosomes during translation can affect both translation rates and protein folding. Different factors can contribute to translational pausing, namely the cis-acting elements of mRNAs [secondary structures, codon context, poly(A) tracts], the chemical modification and the availability of aa-tRNAs, as well as the interactions between the wall of the polypeptide exit tunnel of the ribosome and the nascent peptide.

The prolonged stalling of the ribosome on intact mRNAs or on chemically damaged mRNAs can produce the so-called no-go complex. Ribosomes can also stall on mRNAs which lack the stop codon (non-stop ribosomal complexes). The review from Müller et al., describes in structural detail the various rescue mechanisms which are critical to release both ribosomes and tRNAs sequestered in the no-go and non-stop complexes, to make them available for new rounds of translation.

The sRNA word is very fascinating both for the wide range of mechanisms which control translation and for the questions still open in this field. Carrier et al. review common and uncommon sRNA-mediated mechanisms of translation regulation, mainly focusing on gram negative bacteria. sRNAs can do different jobs, from the classical inhibition of translation by targeting Ribosome Binding Sites (RBSs), to less canonical mechanisms of action which entail base pairing of the sRNA with the 5'UTR or the coding region of the target mRNA, a situation which may favor translation initiation by producing local structural rearrangement on the mRNA.

In *Staphylococcus aureus* antisense small RNAs (asRNAs) are often involved in the control of plasmid copy number, as described in the original research paper of Guimarães et al. The natural plasmid pSA564 produces an asRNA (named RNA1) from a promoter located on the reverse strand of the replication initiation gene (*repA*). As observed for other RepA_N family members, base-pairing between RNA1 and the 5'UTR of *repA* could favor a specific conformer of the 5'UTR which reduces both *repA* transcription and translation. The rapid degradation of RNA1 is probably ensured by the host exoribonucleases RNase J1 and J2.

In conclusion, this Research Topic highlights a selection of original research and review articles representing the current progress in our understanding of the diversity of molecular mechanisms of translation regulation in response to changing environmental conditions or antibiotic treatments. We hope that the readers will enjoy these works as much as we have.

## Author Contributions

AMG and SM have made direct and intellectual contribution to the work and approved it for publication.

## Conflict of Interest

The authors declare that the research was conducted in the absence of any commercial or financial relationships that could be construed as a potential conflict of interest.
